# Supplementary material for: An empirical data analysis of “price runs” in daily financial indices: Dynamically assessing market geometric distributional behavior
Source: PLoS One. 2022 Jul 7;17(7):e0270492. doi: 10.1371/journal.pone.0270492 (PMC9262240; doi:10.1371/journal.pone.0270492)
Supplement: S1 Appendix — (PDF) [file pone.0270492.s002.pdf]

The discrete version of the Anderson-Darling goodness-of-fit test

Determining whether or not a given probability model fits the observed data is one of the most important problems of applied statistics. Even though extensive research has been done regarding this problem, most of it deals with continuous distributions, while studies done for fitting discrete distributions are much less numerous, or at least harder to find.

Discrete distributions, however, are important in many fields as medicine, psychology and engineering. Scientific papers and textbooks often prescribe the chi-square test as the option to use when testing goodness of fit for these distributions.

Nevertheless, it is well known that chi-square tests have some problems, especially when applied to data in which there are bins with just a few entries, and when the expected distribution predicts a very low probability for certain events. Data drawn from a process that can be described by a geometric distribution precisely exhibits both features as it can verified in all the tables shown in previous S1.Appendix.

Bracquemond *et. al.* (2002) [1] review eight alternative methods to the chi-square test and performs a simulation-based comparative study specifically for the geometric distribution. It first consists on checking the empirical significance level against the nominal one for each test, and then performing a power study. They analyzed three tests based on the empirical distribution function, three based on the empirical generating function, the Neyman smooth test, and a test by Nikulin (1992) [2] based on the generalized Smirnov transformation.

The tests that had an overall better performance were the Baringhaus-Henze (BH) test, the Anderson-Darling (AD) test and Nikulin's test. Among these, Nikulin's test was considered to have a satisfying power, but they recommend not to use it for a small samples. The two other tests have the disadvantage of requiring a numerical procedure called parametric bootstrap, that is relatively expensive computationally speaking. However, since the BH test involves by far many more operations than the AD test, we preferred to use the latter in the analysis presented in this paper. The computer power required to carry out the AD tests in this work is reasonable with current technology. The code we wrote to perform the test gives the results within about one second on an ordinary laptop.

On the Anderson-Darling test

The AD test belongs to a family of goodness of fit tests called the Cramér-von Mises tests, which includes the Anderson-Darling test, Watson's test and the Cramér-von Mises test itself.

The family was originally developed to test continuous distributions, but a generalization including discrete distributions appeared for the first time in an article by Choulakian *et.al.* [3].

The principle behind this kind of tests consists in defining a statistic that serves to measure the distance between a theoretical cumulative distribution function $F_0(k)$ and the empirical cumulative distribution function for n events, $\mathbf{F}_n(k)$. Every value of the statistic is associated with a p-value, that can be interpreted as the probability of obtaining a value of the statistic at least as large as the one obtained, given that the null hypothesis

$$\mathcal{H}_0 : \mathbf{F}_n(k) = F_0(k) \tag{1}$$

is true. If the p-value is smaller than a previously defined threshold value α, the null hypothesis is rejected. For the case of the discrete Anderson-Darling test, this statistic is the *Anderson-Darling statistic*:

$$A_n^2 = n \sum_{k=1}^{\infty} \frac{[\mathbf{F}_n(k) - F_0(k)]^2 p_0(k)}{F_0(k)(1 - F_0(k))}, \tag{2}$$

where $p_0 = F_0(k) - F_0(k-1)$. If instead what is being tested is whether the observed data comes from a distribution belonging to a parametric family $F(\cdot;\theta)$, then the parameter θ must be estimated first.

For the case of the geometric distribution $\mathcal{G}(p)$, this is an additional complication, since the distribution of A_n^2, and therefore the correspondence between it and the p-values, depends both on n and on the parameter p. In order to overcome this situation, a numerical technique called parametric bootstrap is used as follows. First, p is estimated using the maximum-likelihood estimator:

$$p_n = \frac{n}{\sum_{i=1}^{n} K_i}, \tag{3}$$

where K_i are the values of the random variable in the sample. Then, a large number of copies of the sample are generated and filled with random numbers taken from the actual geometric distribution $\mathcal{G}(p)$, and the AD statistic is calculated for every one of them. See Fig A. The distribution of the statistic found from the samples can be then integrated up to the value of A_n^2 calculated from the empirical data, in order to find the p-value for our case of interest. For the parametric bootstrap, we used 1000 copies of the sample, which was twice the sample size used by Bracquemond *et. al.* [1] for their tests. As our random number generator, we used the routine *Mersenne-Twister* from the software *Wolfram Mathematica*, which breaks serial correlations to a great extent and has a period of $\approx 2^{19937} - 1$.

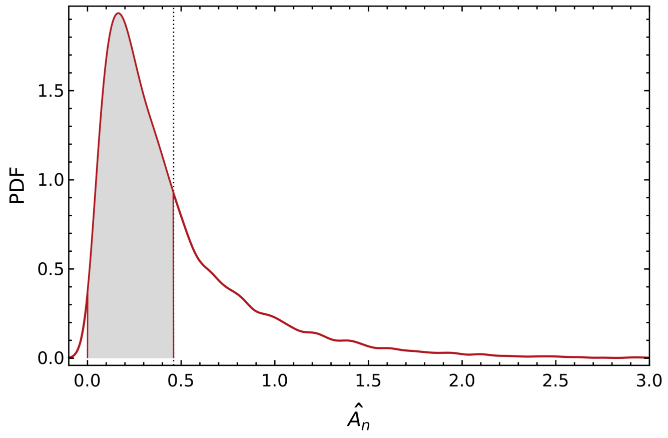

Fig A. Distribution of $\hat{A}_n^2$ generated from 10000 replicas of a sample with $p = 0.502994$. The area under the curve ranging from $[0, p]$ is the p-value that represents the probability of measuring a value as extreme as p. The measured p-value for this sample is 0.609.

References

1. Bracquemond C, Crétois E, Gaudoin O. A comparative study of goodness-of-fit tests for the geometric distribution and application to discrete time reliability. Laboratoire Jean Kuntzmann; 2002.

2. Nikulin MS. Statistic and Goodness-of-fit tests for Grouped Data. Comptes rendus mathematiques de l'Academie des Sciences de Canada. 1992;14(4):151–156.

3. Choulakian V, Lockhart RA, Stephens MA. Cramér-von Mises Statistics for Discrete Distributions. The Canadian Journal of Statistics / La Revue Canadienne de Statistique. 1994;22(1):125–137. doi:10.2307/3315828.
